# Documenting patients’ and providers’ preferences when proposing a randomized controlled trial: a qualitative exploration

**DOI:** 10.1186/s12874-022-01549-1

**Published:** 2022-03-06

**Authors:** Devesh Oberoi, Cynthia Kwok, Yong Li, Cindy Railton, Susan Horsman, Kathleen Reynolds, Anil A. Joy, Karen Marie King, Sasha Michelle Lupichuk, Michael Speca, Nicole Culos-Reed, Linda E. Carlson, Janine Giese-Davis

**Affiliations:** 1 Division of Psychosocial Oncology, Department of Oncology, Faculty of Medicine, Cumming School of Medicine, Department of Psychosocial Resources, Tom Baker Cancer Centre, Holy Cross Site, 2202 2nd St. SW, AB T2S 3C1 Calgary, Canada; 2 Medical Oncology, Tom Baker Cancer Centre, 1331 29 St. NW, AB T2N 4N2 Calgary, Canada; 3 Holy Cross Centre CancerControl Alberta, Alberta Health Services, AB T2S 3C3 Calgary, Canada; 4 Cross Cancer Institute, 1156 University Ave, AB T6G 1Z2 Edmonton, Canada; 5 Long Term Survivors Clinic, Alberta Children’s Hospital, 2888 Shaganappi Trail NW, AB T3B 6AB Calgary, Canada; 6 Medical Oncology, Tom Baker Cancer Centre, 1331 29 St. NW, AB T2N 4N2 Calgary, Canada; 7 Department of Kinesiology, University of Calgary, 2500 University Dr. NW, T2N 1N4 Calgary, AB Canada; 8 Department of Oncology, University of Calgary, 2202 2nd St. SW, T2S 3C1 Calgary, AB Canada

**Keywords:** Breast cancer, Cancer survivors, Transition of care, Cancer care coordination, Telephone survivorship clinic, Oncology nurse

## Abstract

**Background:**

With advances in cancer diagnosis and treatment, women with early-stage breast cancer (ESBC) are living longer, increasing the number of patients receiving post-treatment follow-up care. Best-practice survivorship models recommend transitioning ESBC patients from oncology-provider (OP) care to community-based care. While developing materials for a future randomized controlled trial (RCT) to test the feasibility of a nurse-led Telephone Survivorship Clinic (TSC) for a smooth transition of ESBC survivors to follow-up care, we explored patients’ and OPs’ reactions to several of our proposed methods.

**Methods:**

We used a qualitative study design with thematic analysis and a two-pronged approach. We interviewed OPs, seeking feedback on ways to recruit their ESBC patients for the trial, and ESBC patients, seeking input on a questionnaire package assessing outcomes and processes in the trial.

**Results:**

OPs identified facilitators and barriers and offered suggestions for study design and recruitment process improvement. Facilitators included the novelty and utility of the study and simplicity of methods; barriers included lack of coordination between treating and discharging clinicians, time constraints, language barriers, motivation, and using a paper-based referral letter. OPs suggested using a combination of electronic and paper referral letters and supporting clinicians to help with recruitment. Patient advisors reported satisfaction with the content and length of the assessment package. However, they questioned the relevance of some questions (childhood trauma) while adding questions about trust in physicians and proximity to primary-care providers.

**Conclusions:**

OPs and patient advisors rated our methods for the proposed trial highly for their simplicity and relevance then suggested changes. These findings document processes that could be effective for cancer-patient recruitment in survivorship clinical trials.

**Supplementary Information:**

The online version contains supplementary material available at 10.1186/s12874-022-01549-1.

## Background

Early-stage breast cancer (ESBC) is defined as a disease confined to the breast with or without regional lymph node involvement, and the absence of distant metastatic disease. ESBC patients are living longer [1], increasing the number of patients receiving post-treatment follow-up care [2]. Best-practice survivorship models [3–5] though varied in approach [5], each recommend transitioning non-palliative ESBC patients from oncology-provider (OP) care to community-based care and patient self-management [6, 7]. This success for patients, however, often leads to healthcare-system challenges as the transition of patients to follow-up care involves multiple stakeholders from various disciplines such as medical and radiation oncology, nurse practitioners, and primary care providers (PCPs). One of the challenges in the transition of patients is that communication channels between multi-disciplinary care providers and stakeholders are often limited [8]. Oncologists often report low confidence that patients will receive medically appropriate continuity of care from PCPs [9] and nurses require more time prior to transition to intervene to reduce symptom distress and encourage lifestyle changes [10]. Patients, on the other hand, report uncertainty, fear, depression, confusion, and lack the knowledge and empowerment to self-manage symptoms and surveillance [11]. PCPs are bombarded with specialist follow-up suggestions and have varying comfort with crucial cancer follow-up because many do not receive cancer training [12].

Although completing treatment with a good prognosis seems cause for celebration; instead, some patients report feeling abandoned by their healthcare team [13]. Their cancer-surveillance transition needs are multiple [3] focused on optimizing longer life with the best QOL. These include psychosocial and supportive care [3, 14], surveillance for multiple symptom burden [15] as well as cancer progression, and coaching in self-management to adhere to hormone therapy guidelines and lifestyle changes [16–20]. The optimal model for transitioning ESBC to survivorship is unknown and varies across North America [5, 21]. International guidelines recommend that each patient receive a survivor care plan (SCP) at transition [3] during provider conversations about their specific diagnosis and treatment follow-up. However, a typical SCP [22, 23] is a one-time conversation and limited in empowering either survivors or PCPs over time to manage survivorship care [24]. Randomized trials failing to find significant benefits for this one-time SCP delivery [25, 26], support the need for a short-term transition intervention (3-5 sessions), giving ESBC patients time to learn to integrate self-management skills to improve their survivorship experience.

We may be able to optimize this transfer of care through a theoretically driven telephone or videoconferencing (i.e. zoom) survivorship clinic (TSC), using motivational interviewing. In this process, oncology nurses can provide short-term support conveniently and economically while motivating healthy lifestyles and empowering patients and PCPs.

In previous research we documented that ESBC patients were often left vulnerable during the confusing transition to PCP-care [10]. We conducted a formative study using seed-grant funds to develop a multi-disciplinary team (oncologists, nurses, primary care physicians, psychologists, kinesiologists, and health economists) that also included patient advisors who were themselves at this point of transition. The goal of the team, including the patient advisors, was to develop materials and processes to propose a feasibility trial of a TSC. Together we created a nurse manual for the intervention, a process for training, supervision, and fidelity to the intervention, a design for an initial feasibility trial, recruitment methods, a questionnaire packet with primary and secondary outcomes, and a knowledge-translation plan. This paper reports on two aspects of our foundational work that may prove useful to other cancer survivorship researchers. Because Alberta’s standard of care is to discharge all ESBC patients to primary care, the team developed a provincial recruitment plan and examined how receptive urban and rural cancer clinicians would be to this plan and our recruitment materials. Integrating OPs’ feedback about challenges recruiting ESBC patients at this transition point is crucial. Because patient advisors were key stakeholders, we asked these women to complete the proposed questionnaire packet and provide feedback. Incorporating patients’ feedback to ensure a comprehensive assessment package that reflects their preferences could lead to better adherence to study protocols.

### Objectives

1. To engage with OPs (oncologists, nurse practitioners) to review consent, study objectives, and to obtain feedback on our patient recruitment methods (inclusion/exclusion criteria, patient recruitment flow chart), the feasibility of recruiting patients from their clinics, and suggestions for improvement.
2. To engage with ESBC patients (patient advisors) to explore their views on, tolerance for, and potential contributions to a thorough questionnaire package to assess the major issues faced by ESBC patients in this transition to PCP care post-treatment.

## Methods

### Study design

We used a qualitative study design with open-ended semi-structured interviews for data collection. Interviews were audiotaped, transcribed, and thematically analyzed using inductive thematic analysis to identify themes emerging from the data.

### Participants and procedures

#### Clinicians

 Study-team clinicians (SL, lead for Calgary, and KK, lead for Edmonton) provided a list of 25 breast cancer OPs practicing throughout Alberta. We contacted each OP and provided a study invitation letter and consent form to participate. These clinicians, in turn, recommended others for interviews. In total, we interviewed thirteen OPs. Participants were recruited and interviewed until the research group considered that saturation was reached. The research team conducted one-on-one telephone interviews with each OP to review consent, study objectives, and to obtain feedback on our patient recruitment methods (inclusion/exclusion criteria, patient recruitment flow chart), the feasibility of recruiting patients from their clinics, and suggestions for improvement (Appendix 1).

#### Proposed feasibility-study design

The proposed short feasibility study design presented to OPs included randomization to two active intervention arms: (1) Nurse-initiated TSC; and (2) Patient-initiated TSC. ***Arm 1. Nurse-initiated TSC***: RN would schedule 2 follow-up calls with each patient: (1) within 2 weeks post-discharge/study entry to elicit immediate concerns and deliver an SCP, and at (2) 2 months after initial call to elicit adherence, symptom, and self-management concerns. In this scripted call, the nurse would use motivational interviewing techniques to provide a review of symptoms, respond to follow-up, psychosocial, and adherence-related concerns, present relevant self-management approaches, encourage adherence to medical and lifestyle guidelines, and provide appropriate referral suggestions to the PCP. Nurses record any referral contact. ***Arm 2. Patient-initiated TSC***: The research coordinator would send survivors orientation and reminder sheets 2 times (start, 2 months): outlining the TSC they are eligible to use, possible reasons to call, and encouragement to call the dedicated 1-855 number. Patients were invited to call at any time, leaving a message. The RN would return calls delivering a patient-initiated TSC. The nurse would not review an entire script; rather patients would lead discussions, highlighting specific concerns they would like to review. Arm 2 nurses would have the same script as the nurse-initiated arm and make use of tabbed sections for problems or topics patients initiate. Once a patient describes their concerns, the nurse can skip to the sections most relevant for that call. Nurses record the concern, type of intervention, referrals, duration, and follow-up for each call. In each arm, the nurse would print and send a summary of sessions to patients and PCPs following our standard templates.

#### Patient advisors

 Lead clinicians (SL and KK) referred potentially interested patient advisors from their clinics at the point of discharge to primary care, and the research team contacted them to discuss the study objectives and role of advisors, gauge interest in participating, and to review the consent form. These advisors participated in trial team telephone planning meetings and filled out the questionnaire package to provide feedback.

The inclusion and exclusion criteria for the advisors mirrored the potential randomized patients for the study. Included advisors were (1) women with stage I-III breast cancer at active treatment completion [may return to Endocrine Therapy in the future]; (2) formally discharged or within 0-4 weeks of formal discharge from Cancer Centre to PCP care; (3) within two years of finishing radiation and chemotherapy, whichever came last; (4) English-speaking, 18 or older, and functional status of at least Karnofsky [27] 70 (able to care for self). Exclusion criteria included (1) males and any survivor not discharged to PCP care; (2) patients with significant psychiatric history compromising participation (e.g., major depression for which hospitalization was necessary); (3) unable to perform assessment tasks (e.g., fill out questionnaires, speak on the phone), or unable to care for self; (4) women with stage IV breast cancer; women more than four weeks from discharge to PCP care at the time of recruitment.

We recruited patient advisors living in urban and rural settings to provide an equal representation of provincial healthcare practices. In total, we recruited eight and interviewed five patient advisors, with three dropping out of consideration (one English as a second language concerns, two no time). Our goal was to interview participants until the saturation of ideas was reached. At the completion of four interviews, we felt that that the data was leading towards saturation and at the completion of the fifth interview, the perceived saturation of concepts was achieved. However, as this was a pilot study and we did not have any more candidates to interview, the data collection was ceased at the fifth interview considering that much of the saturation was reached. We asked the advisors to review and comment on study documents intended for the intervention, such as the questionnaire package and nurse training manual, for content breath and applicability to ESBC survivors. Our multi-disciplinary team, including our 5 ESBC patient advisors, created the TSC, training methods, nurse manual survey packet, and recruitment methods.

 Once advisors completed the review of the questionnaire package, we held a one-on-one telephone interview with each advisor to obtain feedback about the assessment materials. The interviews focused on patient advisors’ experience filling out the questionnaires, any problems, and suggestions to increase or decrease domains or questions (Appendix 2). The questionnaire package included demographics, medical history, and response to breast cancer. Additionally, we included questionnaires most relevant to each of 4 perspectives in the multi-disciplinary coordination of transition to primary care – Quality of life [28], Continuity of Care [29], Adherence to Medical Guidelines [10] and Self-reported Activity levels [30]. (Appendix 3). Questionnaires were available through paper or URL via Research Electronic Data Capture (REDCap), an online survey. It also included a short (19 items, 0-6 Likert-type scale, strongly disagree--strongly agree, ten items negatively keyed) custom questionnaire enquiring of their reactions to filling out the questionnaire packet (Appendix 3).

### Qualitative data analysis

Keeping with a realist/essentialist paradigm the inductive thematic analysis allowed for taking an exploratory approach. The initial analysis was performed by the first author (DO) and audited by the senior author (JGD). The first-level coding involved dataset immersion, initial code generation, and development of a flexible code-structure for grouping units into categories. The initial themes that were generated were evaluated by other researchers with regard to the original data. The second-level analysis involved immersion with the preliminary coded data, sorting “semantic” themes representing a patterned response. Codes were discussed by the research team in relation to the interview data in weekly meetings. Themes were reviewed back and forth with the original dataset to ensure fit and intercoder agreement. The final themes retained were those that generated consensus from all team members and were supported by the data. The unanimity was sought for assigning information to the various themes and for collapsing multiple sub-themes to major themes through the process of axial coding. Rigor was supported by full data-immersion, iteration, consensus coding, peer debriefing, reflexive analysis, and audit trial. Data were examined and categorized by using inductive thematic analysis and were coded in the NVIVO v.12 software program (QSR International). Furthermore, the data were supplemented by direct quotes from the participants whenever possible and used to support the themes.

## Results

### Clinicians

#### Participant characteristics

Seven female and six male OPs participated. We recruited seven from tertiary care cancer centres in Calgary and Edmonton, while six were recruited from regional cancer centres in Alberta. Eight of the participants were medical oncologists, two were radiation oncologists, and three were nurse practitioners.

### Themes

When interviewed, OPs across Alberta endorsed the project, committing to recruit for it. Each reported that patients during discharge feel anxious and lost. Additionally, they indicated that some survivors experienced greater survivorship needs that may not be identified during treatment at the cancer-center and may complicate community-based care. After grouping relevant codes together, three main categories emerged about the ease of use of recruitment materials and strategies: *Facilitators to recruitment*, *Barriers to recruitment*, and *Suggestions for improvement in recruitment methods*.

### Facilitators

#### I. Value of the study

The value of the study emerged as a major theme, with three sub-themes. OPs’ views were that the study was desperately needed, inclusive, and our methods were simple for them to implement.

##### a. The study is imperative and desperately needed

Clinicians were excited and optimistic about the study. They mentioned that patients were often anxious at discharge for fear of losing contact with their healthcare providers and anticipated that the proposed trial would come as a great relief and reassurance to many of them.

Clinicians working at regional cancer centres further added that such centres were often equipped with fewer resources than bigger cancer centres and welcomed the study. One of them stated that patients’ anxiety at the time of discharge was a battle, not just for patients but for the entire clinical staff at the centre, especially when patients did not have a family doctor. He also anticipated that follow-up support after discharge would improve patients’ adherence with hormonal therapy “*If they know that they're part of this or somebody is calling them, they're actually taking their pills at that point of time*.” (Participant 2, medical oncologist, regional cancer centre (Quote 1.1a, Table 1).

**Table 1:** Quotes from oncology providers

	b. Study is inclusive in nature	1.1b “*The recruitment methods I think are inclusive for basically capturing any patient that may be interested in the study because this is also through nurse practitioner and so I think the – I think it's inclusive of all patients who – no matter what they've gone through in terms of treatment*: - Participant 5, medical oncologist, tertiary cancer centre
	c. Simplicity of the methods	1.1c “*And I guess what appeals to me is that you seem to have a simplified recruitment process so that it won't take a lot of our time*”. Participant 6, med onco, regional 1.2c “*I mean yeah, I mean the fact that we can email you guys the contact information, you'll take care of the rest, that's just – it's great*.” Participant P8, medical oncologist, tertiary cancer centre
2. Barriers Health system barriers	*a. Coordination between discharging clinicians*	2.1 a “*Having an awareness of – for example between clinical and nursing and us, when they are booking their discharge, they could put the sheet on there”.* Participant 5, medical oncologist, tertiary cancer centre 2.2a “*The offer needs to be made in the clinic by myself and the nurse that's working with me in the follow-up clinic*.”Participant4, Radiation oncologist, tertiary cancer centre 2.3 a “*It'll be easy for the ones that don't get radiation or don't need radiation, they just see us and if they come back in like three months or two months to make sure you're doing okay on the hormonal therapy and then I discharge them, those will be straightforward*” Participant 2, medical oncologist, regional cancer centre 2.4a “*Whoever is the last to see or treat, so it often falls on the radiation – patients undergoing radiation, it will often fall to the radiation oncologist to discharge. Often it's just through the chart that, you know, I'll see my patients for the last time for me and I see that radiation oncology still has an appointment down the line so I could certainly in my last note on the patient indicate that they'd be a good candidate and even write that in the chart.”* Participant 6, medical oncologist, regional cancer centre
	b. *Time constraints for clinicians*	2.1b *“So, for me, it's – for the time sometimes, it's just in some cases, because I am the sole oncologist here in these areas. So, it's just sometimes I get too busy and might not have always the time to discuss that*.” Participant 7, medical oncologist, regional cancer centre 2.2b *You can prepare as best you can but if you have a very busy clinic talking about trials, interventions, studies does take more time and it can fall into the backseat if you will*”. Participant 12, medical oncologist, tertiary cancer centre
Patient related barriers		2.1 c “*And that's the only thing is that lots of my patients may be able to speak English but they may not be written-language literate so for them they may find some barrier to understanding the information that they would be given but they are obviously not going to be eligible*” Participant 7, medical oncologist, regional cancer centre 2.2c “*They might work for some but like most of them like mails rather than email. The only issue like the response rates, I am not sure about the response rate*.” Participant 2, medical oncologist, regional cancer centre 2.3c “*You know obviously I will expect the uptake to be lower than those who receive phone call directly from the program; some of them are elderly patients, they may not remember this and/or the younger patients are getting on with their lives and going back to work*” Participant 7, medical oncologist, regional cancer centre
Study related barriers	a. Referral materials	2.1 d “*Honestly I think that's too wordy and I know that sounds so placating but people will probably not read it*.: P1, nurse prac, tertiary cancer centre 2.2 d “*when they are bored waiting for me often and they’ll read whatever is in the room and two, I can just point and they can take a picture*. “Participant 1, nurse prac, tertiary cancer centre
3. suggestions for improvement	*a. Combination of electronic and paper letter*	3.1 a “*And I agree, have it there and then if I want to print out seven copies and have it in my binder that helps me, well, off I go, right. You shouldn’t provide those, people can print them themselves if they need*.” Participant 4, Rad onc, tertiary cancer centre 3.2 a “*Our patient department is all in one very tiny but all in one area. So having a paper letter to give to patients I think in some ways is quite practical. There tends to be less use of shared drive access to forms here so if you were going to use that as a place to store something on the shared drive, that would have to be really well communicated*” Participant 7, medical oncologist,regional cancer centre
	b. support for clinicians	3.1 b “*I think she would be on board for this because she does a lot of the survivorship here anyways. And I think would be the one that gets you a lot of patients*” Participant 2, medical oncologist, regional cancer centre 3.2b “*And I think even just a short letter for them with – even with just the eligibility criteria and then they could just remind us of it.”* Participant *5, med onc, tertiary* cancer centre 3.3 b “*I've had many a times somebody sitting in clinic and they would identify the potential eligible patients and say doctor, when you're done can I approach the patient? Absolutely, that makes recruitment so much more effective and easier*.” Participant 12, medical oncologist, tertiary cancer centre
1. Facilitators	a. Study is imperative and desperately needed	1.1a “*Some of our patients don't have the family care doctors and there is a lot of anxiety that comes along with the breast cancer patients too. So it would be nice to kind of see if it [intervention] makes any kind of difference whether you give them a number to call versus a nurse calling, looking at the outcomes from that perspective as well too*.” Participant 2, medical oncologist, regional cancer centre 1.2a “*Just giving them an opportunity to participate in the study which would perhaps change their perception of what happens at the end of care. I think it's really quite valuable because I hear and I see on their faces a great deal of fear of the unknown and comfort with what they have*.”- Participant 11, Nurse practitioner, tertiary cancer centre 1.3a “*Well and ultimately the study itself, you know, it is certainly novel and interesting to understand what's the patient's perception in terms of follow-up care, so that the question that the study question is very relevant and very topica*l.” Participant 12, medical oncologist, tertiary care centre 1.4a “What appeals, I don't know, the recruitment process seems to be relatively straightforward. I guess what appeals to me is we are really looking forward to – looks like finally we're going to be able to get some clinical trials going in the community centres and so we're all for research.” Participant 6, medical oncologist, regional cancer centre

Another clinician echoed similar views and mentioned that she was always anxious about patients’ follow-up care following discharge, and this study was a welcome move. She anticipated that the mere opportunity to participate in the study could change patients’ perceptions of their follow-up care. She added that patients were often anxious about what is going to happen when they are released to someone for whom “oncology is not their forte.” (Participant 11, Nurse practitioner, tertiary cancer centre). (Quote 1.2a, Table 1). Clinicians also felt that offering patients this study and allowing them the choice to participate would give them a say in their follow-up care. This additional power in the doctor-patient relationship might be useful for their overall wellbeing. One of the participants mentioned that the usefulness of the study was nested in its patient-centred-care model. She added that at times the actual care is lost during the provision of care and having a real conversation with patients to understand how they are coping with their illness was a step in the right direction. Lastly, participants also felt that the study was novel and interesting. It would facilitate the healthcare professionals' understanding of patients’ perspectives of their discharge and follow-up care (Quote 1.3a, Quote 1.4a, Table 1).

##### b. The study is inclusive

One participant stated that the study was inclusive of all patients that may be interested in participating, regardless of what treatment they have received or for how long. Similar views were echoed by other participants as well. One of them believed that patients would be flattered to be invited to this study, as it was a way for them to participate in their own follow–up care. Another participant was impressed with the broad inclusion criteria and was happy that the study was available and accessible to all breast cancer patients (Quote 1.1b, Table 1).

#### II. Simplicity of the methods

Overall, OP’s expressed the general consensus that recruitment methods were simple and very straightforward, “without too many detours”. Ease of recruitment and simplicity of the methods was a great facilitator for most OPs. Most participants mentioned that the recruitment method was “*good and easy*”- P2. For them, the most attractive part was that they were simply required to redirect patients to the research team, who would “take care of everything”. One participant mentioned that he would be seeing the patients in his review clinic post-completion of treatment, and when the patients are ready to be discharged to the family doctor, he would inform them about this study and offer them participation. Another clinician echoed similar views and added that the process of informed consent and other steps of the study was how most of the clinicians would have wanted it to be (Quote 1.1c, Quote 1.2c, Table 1). One of the clinicians was excited that this study was being implemented at the end of treatment, when there were no other competing trials.

### Barriers

Barriers in recruitment/implementation of the study.

Three factors emerged including i Health-System-, ii patient-, and iii study-related barriers. Each of these allowed the study team insight into the often confusing, multi-layered treatment system for breast cancer patients in the province.

#### I. Health-system-related barriers

These barriers were about the often-difficult coordination between the discharging clinicians and time constraints for clinicians.

##### a. Coordination between discharging clinicians

A striking outcome of these interviews was learning the many differing ways OPs work with their patients, and ESBC patients’ many different paths through the healthcare system. Patient paths differed within tertiary versus regional centres, and from clinician to clinician. Seven of thirteen clinicians mentioned that once patients completed treatment in their department, they would either move to another department (such as radiation oncology) or were often discharged to the PCP by a nurse practitioner rather than them (Quote 2.1 a; Quote 2.2a, Table 1).

Overall, they expressed a general consensus that better coordination between medical oncologists, nurse practitioners, and radiation oncologists within the cancer centres would facilitate recruitment of participants for the study (Quote 2.3 a; Quote 2.4 a; Table 1). Clinicians agreed that the barriers to recruit patients for the trial had little to do with researchers, who laid out a straightforward plan, and more to do with clinicians, who needed to plan and coordinate better on behalf of their patients. Their suggestion for the research team was to educate clinicians at the medical centre about the whole process (Quote 2.5 a).

##### b. Time constraints for clinicians

OPs reported time constraints related to busy clinics, high patient loads, staff shortages (particularly in regional cancer centres), and the many active studies open for recruitment. One participant mentioned that some appointments got pushed or delayed by 30 min, and clinicians often “*did not have those five minutes to have this conversation*.” *(Quote 2.1b;* Quote 2.2b, Table 1).

Time was a crucial factor, especially with high patient loads, and if recruiting patients was time-consuming, they would rather not do it. A few clinicians also highlighted that although time was a constraint, the process of recruiting patients was easy and non-time-consuming. One of the participants mentioned that it would make the process less onerous if they “worded it in a way” to convey that the research team would be able to provide more information to interested patients.

#### II. Patient-related barriers

##### Language barriers

Clinicians identified patients’ language skills as a barrier. One clinician mentioned that although most of her patients spoke fluent English, many did not have adequate reading skills; hence, the little time available during the consultation plus lots of reading materials for patients could make recruitment for the study less efficient. She also suspected that unless the language eligibility criteria were relaxed, some of the participants would be ineligible to participate (Quote 2.1 c). Clinicians suggested translating all study materials into other languages like Cantonese to cater to the needs of non-English-speaking patients.

Another patient-related barrier was the ageing population who had somewhat less inclination to use cell phones or answer electronic surveys. This was not identified as a significant problem after the research team reminded the clinicians that participants could complete mailed questionnaires.

##### Lack of motivation

 Another barrier for study uptake was patient’s potential lack of motivation to make phone calls themselves, rather than having someone from the program call them (one arm of the study required participants to initiate calls). Clinicians believed that most patients were elderly and that younger patients tended to move on with their lives, hence the motivation and, subsequently, the uptake could be less. One clinician anticipated patients’ reluctance to participate in the program as the only barrier and emphasized that if the patients agreed to participate in the study, there would not be any additional barriers (Quotes 2.2 c; 2.3c, Table 1). One OP was concerned with the length of the referral letter and suggested to shorten it. She suggested that the letter be no more than one page long and in point form, so it is easier to read.

### Suggestions for improvement

#### a. Combination of electronic and paper letter

Participants’ views were divided between using a paper referral letter alone or using both paper and electronic referral letter stored on shared computer drives.

One clinician mentioned that he would not like to carry multiple copies of the letter in a binder, but if the letter was saved on a shared computer drive, anyone could easily print it out when needed. He also added that if there was any change in consent form or study protocol, the clinicians would be able to print the latest version from the shared drive (Quote 3.1 a). However, many other clinicians, especially those from smaller cancer centres, were happy to use paper letters alone. One clinician mentioned that if she knew where to find the referral letter, it didn’t matter to her whether the letter was stored on a shared drive or printed copies were stored in the office (Quote 3.2 a).

A few clinicians suggested that they should be provided with both electronic and physical copies of the letter, which would facilitate the recruitment process. They would be able to provide physical copies to all healthcare providers, including nurses, as well as print the letter on the spot for patients at the time of discharge. Another suggestion was to have study posters in the clinic which the patients could read as they waited for their appointment or could take a picture of the clinicians’ instructions. Clinicians expressed a consensus that researchers should place study posters at strategic, easily accessible locations. Posters would allow clinicians to save time, making the process more efficient.

#### b. Support for clinicians

Some clinicians mentioned that as they were limited in time and resources, it would be useful for them to have support in helping to recruit participants. One clinician emphasized the need to bring nurse practitioners on board to help with recruitment as the two nurse practitioners on his team were primarily involved with cancer survivorship programs (Quote 3.1 b). Another clinician concurred with this view and added that it was imperative that the clinic nurses also had a copy of eligibility criteria to aid in recruitment. She advised about the need to draft and circulate a short letter and a copy of the eligibility criteria to the nurses so that they were aware of this trial (Quote 3.2 b).

 One participant also requested constant reminders from the research team to help them keep an eye on patients who were close to discharge while another participant mentioned it would be very helpful to have a research student sit in the clinic and identify potentially eligible participants (Quote 3.3 b).

Lastly, one of the clinicians suggested adopting a systems-based approach rather than an “individual practitioner making referrals” approach. He added that if the clinicians could be provided with a weekly or a monthly download of “No further recall (NFR)” orders (in other words, discharged) associated with the cancer diagnosis, they would just pass that to the researcher for contacting eligible patients.

### Patient interviews

#### Participant characteristics

A total of five patient advisors volunteered after specific referrals of 8 ESBC patients. They were all Caucasian females and had two children each. Most of patients (3) were married, one was divorced, and one widowed. The patients’ education varied from high school to a master’s degree. The mean age was 55.98 ± 5.54 years. Two participants were employed full-time, one part-time, with two retired. Two had been diagnosed in 2014 and 2015 each and one in 2016 with stage 1 (*n *=2) or stage 2 (*n *=2) cancer. All five patients had received surgical treatment, four received chemo and radiation therapy, and three received hormonal treatment.

Patient advisors anonymously completed our questionnaire packet (URL/paper), and when interviewed, did not advocate shortening it to reduce time to completion (M =1.5 h). They requested a “trust in physician scale.” Patient responses were classified into two broad themes: (a) overall experiences with the assessment package, (b) problems associated with the questionnaires.

They completed a custom questionnaire asking for feedback about their experience filling out our questionnaire packet for the proposed RCT. Despite the length of the questionnaire packet (on average, it took advisors 91.8 min to complete: range 74-134), they rated the custom questionnaire positively (Mean 80.64 (SD = 12.72). The most strongly agreed to items had medians of 5 (8 items) with 0-6 strongly disagree—strongly agree: Examples, I was satisfied with the questionnaires (Range (R)=5-5), I think the content of the assessment package will be helpful (*R *=4-6), The assessments helped me to communicate what I am feeling post-treatment (*R *=3-6), These questions will help this team understand how cancer survivors feel post-treatment (*R *=4-5). Patients most strongly disagreed with four items: It was difficult to understand some instructions (*R *=1-4), I often felt confused filling out the questionnaires (*R *=0-4), it was difficult to understand some instructions (*R *=1-4), and I felt upset as I was filling out the questionnaires (*R *=0-1). Two items with the highest standard deviations (1.817 and 1.789 respectively) were further discussed during the interview process: Some important post-treatment experiences were not included in the questionnaires (*R *=0-5), I am not sure why some of the questions were asked (*R *=0-5).

#### Overall experience going through the assessment

##### a. Access to information needed to answer the questions

All patients mentioned that they had records of medical details at home to answer diagnosis and treatment questions in the assessment. One participant mentioned that having the information was empowering and helpful in understanding her own specific case. Another patent mentioned that she was happy to be asked her patient identification number, so in case she was unable to provide any piece of information, researchers could pull information from her medical records. Likewise, another patient mentioned it was helpful to have her husband by her side while answering the assessment, so in case she did not have a piece of information, her husband could help her recall some of the information; but overall, there was nothing asked in the assessment that they didn’t know (Quote 4.1a, Table 2). One patient, who was unable to remember all the information needed to answer the questionnaires, attributed it to her “chemo brain.” However, her overall experience in answering the questionnaires was good.

**Table 2:** Quotes from patient advisors

Overall Experience	a.*Access to information needed to answer the assessment*	4.1a “*I don’t think there's anything that I didn’t have an answer to*” Participant 1
	b. content	4.1 b “*Many of the items just did not seem relevant to me*”. Participant 2 4.2b “*And I think you just kind of, push it to the back of your mind and continue on your normal daily activity as much as you can so that too t the questionnaire made you stop and think, oh, yeah, right. So, I did find it helpful*.” Participant 3 4.3 b “I just know I did it online and everything was clear to me so I just answered it the best I could”. Participant 2 4.4 b “*I can't really think of anything that could have made it better*” Participant 4 4.5 b “*Oh, I mean it was good. You know, it was totally understandable and it was easy to go through and – yeah.*” Participant 4
	c. length of the package	4. 1c “*I didn't find it that overwhelming, that I thought it was really quite easy and straightforward so I didn’t – I can't think of anything that really shouldn’t be missing*” Participant 3
Problems with the questionnaire	*a. Relevance of questions*	5.1a “*I don't understand the connection but I'm sure somebody does. There are smart people out there I'm sure they understand the connection*.” Participant 5 5.2a “*You can't quantify like you can't give a hard number for some of the information that you're looking for if it is more perception*.” Participant 1 5.3 a “*I think they struck the right tone in terms of the information that you're trying to get and how they asked the questions*.” Participant 1 5.4 a “*we don’t have in the questions in front of me now. It’s a little difficult to answer that but I would say for the most part I don’t think there's any area that you can eliminate*. “Participant 1
	b *Missing domains*	5.1 b “*The trust in your primary care like my original doctor, you know, and I had a harder time finding a new doctor. I saw one first and yeah, I didn't really care for her. So, anyhow I found this guy and he is better but I think time will tell. I’ve only seen him a couple of times so I think in time it’ll tell and you know, how much more comfortable you can be*.” Participant 5
	*c. Timing of administration*	5.1 c “*What's happening right then, might be more relevant that things will brush off after a month you’re trying to forget about because life gets in your way but something happening during that time*”. Participant 5

##### b. Content

Participants did not have any issues comprehending the content of the assessment questions. They mentioned the questions were easy to understand, although they acknowledged difficulty with questions specific to tumour characteristics, making them go back to their medical records to double-check information. “Besides the childhood questions [Childhood Trauma Questionnaire], I think most of them were on point. So, I didn’t find anything terribly bad about it.” One of the participants raised concerns about the relevance of some of the questions (Quote 4.1 b, Table 2).

Participants mentioned that often people like to forget about their cancer journey and move on in life (a preference which may adversely affect participation in follow-up plans). However, this assessment allowed them to pause and reflect on their cancer experiences and what they had gone through. They added that they found this reflection most helpful. In particular, the fear of a cancer recurrence questionnaire was rated as a highly useful component of the assessment: in a participant’s words, “because I think everybody has that” (4.2b, Table 2). Another aspect of the assessment that was rated highly by the participants was its simplicity and the opportunity to complete it online in one or multiple sittings. (Quote 4.3 b). “I just know I did it online, and everything was clear to me, so I just answered it the best I could.” Participant 2.

##### c. Length

Participants were divided in their opinions about the length of the assessments. Three of them felt that assessments were too long or too many questions, and some reported that they were exhausted after completing the questionnaires or had to take breaks in between. One suggestion was adding a line at the beginning of the questionnaire instructing the participants about how long it would take them to finish the assessment to give them a heads up. The other two participants were quite comfortable with the length of the assessments and did not think it was too long or difficult to fill out. One of them said she finished the entire assessment in one sitting (Quote 4. 1c, Table 2).

#### Problems with the questionnaire

##### a. Relevance of questions

Some participants were not exactly sure about the relevance of some of the questions on the assessment, and the connection of those questions with their cancer, particularly the “childhood [trauma] things.” However, they anticipated that the researchers, being “smart people,” would be able to find some connection. Likewise, another participant wondered why she was being asked certain questions and felt that some of the questions were duplicate and aimed at eliciting similar information (Quote 5.1a). Some patients reported difficulty assigning a number or a score to some of the questions. They added that the score would vary as per their situation (Quote 5.2a). Most participants did not feel the need to eliminate any domains or questions from the assessment. However, one participant had a slightly opposing view and mentioned that participant demographics and medical history could be eliminated from the assessment as she did not feel they were important enough in the context of the assessment (Quote 5.4 a, Table 2).

##### b. Missing domains

**Medical**

One of the participants reported that patients’ proximity or the distance to their PCPs was missing from the assessments and that would be more important to patients following discharge. Another patient suggested that the questionnaires ask how the patients were feeling on a daily basis. According to her, such information would help the nurse get the right idea of the patient’s ongoing condition and would enable her to call and have a discussion with the patient and maintain a record of it. One participant suggested adding “trust in the physician--oncologist, family doctor,” to the assessment. The participant narrated her ordeal of having to find a new doctor as her previous doctor had moved away. She added it took a bit of time for her to adjust to her new doctor and to be able to trust his care. In cancer care, they have to be able to trust their healthcare providers and know that their healthcare providers trust the patients’ judgment as well (Quote 5.1 b, Table 2).

##### c. Timing of administration of the assessment

 Two participants mentioned they would have appreciated the administration of the assessment while they were undergoing treatment as things would be fresh in their minds, and their responses would have been more accurate. They mentioned their experiences and views might be more relevant as they were undergoing treatment than it was a few months later. Another participant suggested that it would be helpful to have these assessments at multiple time points, first one during the treatment phase, and then later (Quote 5.1 c, Table 2).

## Discussion

In this two-pronged qualitative study, we interviewed OPs’ for feedback on a draft recruitment protocol across Alberta. OPs included medical oncologists, radiation oncologists and nurse practitioners from both tertiary and regional cancer centres. We also asked ESBC patient advisors to complete a questionnaire packet, just as they would in the trial, and provide feedback in interviews about their experience. The proposed RCT this study prepared for sought to test the effectiveness of a TSC intervention for facilitating the smooth transition of ESBC patients to primary care following treatment. Although the success of RCTs fundamentally rests on the ability to recruit participants and retain their adherence to completing assessment protocols, researchers often do not take time to hear directly from patients and providers prior to setting up protocols. Due to a small seed grant, we examined patients’ and providers’ preferences so as to make protocol changes.

Outcomes of this study reassured us that parts of our draft protocol satisfied each group, OPs and patients. However, other aspects surprised us and are worthy of the attention of other researchers. First, clinicians were excited and relieved to hear of such a trial and to imagine their ESBC survivors’ relief receiving short-term survivorship transition care in either arm of the proposed feasibility trial. They acknowledged that at the time of discharge to primary care, ESBC patients are anxious about their continuity of care. This is consistent with past studies highlighting elevated anxiety and depression in some of the cancer patients at the time of discharge [31, 32]. In this backdrop, the proposed RCT is critical to facilitate a smooth transition of care. Their response likely indicates that Alberta’s protocol to discharge to primary care after treatment creates anxiety not only for patients but for providers. Given the straightforward referral of patients to the research team, the OPs found our recruitment process not time-consuming, anticipating that it would fit into their regular consultations at discharge. They found it helpful that the study was inclusive of all ESBC patients, regardless of their treatment type.

Secondly, the perceived lack of coordination between different OPs (medical and radiation oncologists, clinic nurses, nurse practitioners, and pharmacists) created barriers not only for the study but also for smooth transitions for patients. The findings corroborated past research on the lack of care coordination between oncology providers [32]. Clinicians stated that in most cases, once patients finish chemotherapy and move to radiation therapy, or vice-versa, they would have little information about patients’ discharge times and plans. For providers, the enormous variability in how each clinician structured ESBC patients’ paths through treatment to discharge changed fundamentally how we imagined and needed to tailor recruitment. Specific treatment protocols targeted to breast cancer tumour type and stage also determined patients’ paths. Tertiary centres managed these paths differently than regional centres. Differing personnel (medical and radiation oncologists, nurse practitioners, and pharmacists) handled aspects of discharge and would need information and training to recruit these patients.

Improving uptake from OPs’ perspectives, including shortening the referral letter and putting it on the shared hard drive so that they could choose to carry hard copies or print from the many locations in which they saw patients. Sometimes clinics were extremely busy, and consultations ran over time, making it difficult to recruit participants. Clinicians added that having a research assistant sit in clinics would facilitate coordination and recruitment. Another suggestion was to involve nurse practitioners in the recruitment process, as they were often the ones responsible for discharging ESBC patients to primary care. The role of clinical trial nurses in recruitment and the conduct of oncology clinical trials has been documented in past studies [33]. OPs, particularly in regional cancer centres, regretted that the protocol excluded non-English-speaking patients and indicated that for those who spoke English as their second language (ESL), many could not read English written materials. This is consistent with past research highlighting low English fluency levels in patients as a barrier to recruitment and warranting institutions to explore more effective training opportunities for research staff to engage interpreters and adopt recruitment and study materials in multiple languages [34]. Additionally, their feedback changed some of our inclusion/exclusion criteria and broadened the timing of recruitment, training for recruitment plans, and recruitment personnel to work within these varying structures. In response to this feedback the study team altered the materials and methods to clarify inclusion/exclusion criteria, broaden our recruitment models to include paper and digital recruitment materials, and specify the flow of recruitment based on tertiary versus regional cancer-center location.

For patients, we found that they tolerated the 90-minute assessment well. Some of the participants found the package lengthy and suggested adding a sentence at the beginning of the package warning participants of the total time to completion. Some patients felt reassured by the many domains of questionnaires, reporting that “we got it” about what they were experiencing. They reported having a pleasant experience completing the questionnaires saying it allowed them to reflect on their cancer journey. We were concerned that they might not have enough information at home to answer questions on medical aspects of their diagnosis and treatment; however, they mentioned they had all the information required. It was important for them to have the flexibility to complete the questionnaire in single or multiple sittings, both online or offline.

Patients were helpful in suggesting changes to the questionnaire package. Several asked that we include questions about how much they trusted their family physician and oncologist. This important aspect of survivorship is rarely tapped in large-scale RCTs, yet these patients discussed how their wellbeing in survivorship depended heavily on this trust. A trusting relationship between patients and physicians results in facilitated communication, reduction in patient fear, better decision making and improved treatment adherence [35]. Patients suggested that adding questions related to their day-to-day health, in diary form, would add value to the questionnaire package.

However, many did not understand why we were asking about childhood trauma. A history of childhood trauma often predicts disease course and all-cause mortality across illnesses. Including these questions may be important in future clinical trials, yet their feedback allowed us to realize that we needed to prepare them to expect these questions before sending the questionnaires. For some participants, the administration of the questionnaires, both during and post-treatment, would improve the study, a study design we had not considered but might improve continuity of care. Lastly, their universal endorsement of the fear of recurrence items highlights the importance of this construct for ESBC survivors. In response to this feedback, our team added a physician trust scale to our process measures, we also included distance from patient to physician. The team also committed to better prepare patients for the childhood trauma questions within the packet.

### Methodological implications for the trial design

#### For oncology providers

In general, methods for researching the ongoing difficulties cancer survivors may face are often complicated by oncology clinic discharge (10, ). Locating survivors years after discharge either relies on cancer registries or a continuity of records within oncology centres that often does not exist. In the current study, we revealed some difficulties of recruiting survivors at the point of discharge even while they are still in oncology clinic care. Suggestions from oncology providers can improve methods for recruitment. They suggested that to be successful, researchers may need to navigate the many practitioners breast cancer patients may see over the course of their treatment to locate who will discharge them. They offered suggestions to facilitate having recruitment materials handy for clinicians in the fluid meeting locations necessary particularly in regional cancer centres. They also streamlined both the inclusion/exclusion criteria checklist for clinicians and invitation letters for patients to reduce their time commitment. These suggestions can improve survivorship researchers’ methods for working with clinicians.

#### For patients

Likewise, our assessment package was rated highly by our patient advisors. Although survivorship research usually includes a set of standardized questionnaires, researchers rarely ask patients for feedback on their questionnaire packages. However, the current study makes clear that for our patient advisors none of the standardized questions addressed a few crucial underlying aspects of their ability to fare well in survivorship. In this case, trust in their physicians (oncologists and primary care physicians) felt pivotal to them. Likewise, how well they could fare with discharge to primary care physicians may depend on how close those PCPs were to where they lived. As this was a provincial study many of these survivors live rurally sometimes hours away from their PCPs. These contributions from patients can inform future methods in survivorship research seeking to engage patients outside of urban tertiary centres.

#### Limitations

Our clinician panel included various cancer care providers such as medical oncologists, radiation oncologists, and nurse practitioners. We did not seek saturation of ideas in each of these subgroups. Similarly, the oncology providers were from regional cancer centres as well as metropolitan tertiary cancer centres, and we did not seek saturation of ideas within each subgroup. Our sample size of patient advisers was relatively small for normally estimated data saturation. It is possible, with an increase in sample size, new themes may emerge. 

## Conclusions

 Our recruitment methods for the proposed randomized trial of a TSC for ESBC patients to facilitate improved follow-up care were rated highly by the OPs and patient advisors, and feedback from each can improve methods for survivorship research.

## Supplementary Information

**Additional file 1.** Appendix.

## Data Availability

The datasets used and/or analyzed during the current study are available from the corresponding author on reasonable request.
