# Supplementary material for: Documenting patients’ and providers’ preferences when proposing a randomized controlled trial: a qualitative exploration
Source: BMC Med Res Methodol. 2022 Mar 6;22:64. doi: 10.1186/s12874-022-01549-1 (PMC8898414; doi:10.1186/s12874-022-01549-1)
Supplement: Supplementary file 1 — Additional file 1. Appendix. [file 12874_2022_1549_MOESM1_ESM.docx]

**Appendix 1: Interview guide for clinicians**

1. I’d like to start by asking you what appeals to you about the recruitment methods we have described.

2: What difficulties do you foresee in using these methods to recruit your patients from your clinic? (If appropriate, ask for elaboration.)

3: What changes would you like to see to make this easier for your clinic?-(if yes) How would these changes make it easier?”- (if no). Could you say more about that please?

4: What other barriers would there be for you in referring your patients to our study?

5: Is there anything else you would like the research team to know?

**Appendix 2: Interview guide for patient advisors**

1. I’d like to start by asking you to tell me what your experience going through this assessment was like overall during this study.

2: Were there any problems for you with these questionnaires? (If appropriate, ask for elaboration.)

3: What aspects of this assessment did you like the best?-(if yes) what was most helpful?” (Ask for elaboration as appropriate.)- (if no) Could you say more about that please. (Ask for elaboration as appropriate.)

4: What could have made your experience with the assessment package better? (For each suggestion, give prompts for elaboration.)

5: Is there anything else you would like the research team to know?

These questionnaires and assessments tap into some main domains that we think are important for survivors and that might change over time with intervention. We have sent you a list of these domains so that you can look them over and think about them as they relate to your own experience and the experience of other women you know who have gone through breast cancer.

6: What domains might be missing that you feel would be important to capture?

7: If you were going to eliminate a domain to shorten the assessment time, what would that be?

**Appendix 3: Questionnaire Package completed by Patient Advisors**

We included questionnaires most relevant to each of 4 perspectives in the multi-disciplinary coordination of transition to primary care.

1. **Patient--Quality of Life: Primary Outcome:** The Functional Analysis of Cancer Therapy Breast: Breast-Cancer Specific Subscale. This outcome is commonly used in BC studies, including our own (37,38)and is reliable and valid.
2. **Oncology Provider--Continuity of Care: Primary Outcome:** Patient Continuity of Care Checklist(39). This is a reliable and valid short questionnaire developed in Canada that has been used with colorectal cancer patients(29). We used a modification of the short version as per focusing on post-hospital discharge.
3. **PCP--Adherence to Medical Guidelines (subjective and objective): Primary Outcomes: Patient-Reported Clinical Exam, Mammography, and ET adherence.** We used patient report of adherence to clinical exam, mammography, and ET adherence found to mirror closely chart review and pharmacy database records in our previous trial(10)l.
4. **Nurse--Healthy Lifestyle and Symptom Management: Primary Outcome: Self-reported Activity levels.** We used the modified Godin Leisure-Time Exercise Questionnaire(30)to include aerobic and resistance PA levels.The GLTEQ is the most widely used self-report measure of PA levels in cancer patients(40). The leisure score index (LSI) provides a standardized measure of PA levels, allowing for adequate classification. Multiplying the frequency and duration of each mild, moderate and vigorous activity and adding these together provides total weekly PA minutes. Used with adult cancer patients and survivors, it is reliable and valid compared with nine self-report measures of exercise(41). To determine adherence to PA guidelines (150 min of moderate, or 75 min of vigorous activity), total weekly moderate activity is added to two times vigorous activity mins.(42)
